# Lyme Borreliosis, Po River Valley, Italy

**DOI:** 10.3201/eid1608.100152

**Published:** 2010-08

**Authors:** Dario Pistone, Massimo Pajoro, Massimo Fabbi, Nadia Vicari, Piero Marone, Claudio Genchi, Stefano Novati, Davide Sassera, Sara Epis, Claudio Bandi

**Affiliations:** Fondazione Istituto di Ricovero e Cura a Carattere Scientifico Policlinico San Matteo, Pavia, Italy (D. Pistone, P. Marone, S. Novati); Istituto Zooprofilattico Sperimentale della Lombardia e dell’Emilia Romagna, Pavia (M. Pajoro, M. Fabbi, N. Vicari); Università degli Studi di Milano, Milano, Italy (C. Genchi, D. Sassera, S. Epis, C. Bandi); 1 These authors contributed equally to this article.

**Keywords:** Borrelia afzelii, Borrelia lusitaniae, suburban areas, Italy, ticks, vector-borne infections, bacteria, zoonoses, dispatch

## Abstract

We aimed to determine the presence of *Ixodes ricinus* ticks in heavily populated areas of the Po River Valley after report of a Lyme disease case. Eighteen percent of ticks examined from 3 locations were positive for Lyme disease borreliae. Lyme disease was diagnosed for 3 workers at risk for tick bite.

Lyme disease, caused by *Borrelia burgdorferi* sensu lato (*1*), is endemic to various areas of Italy (*2*). The main vector of Lyme disease in Italy is the hard tick (*Ixodes ricinus*), a species widespread in mountain regions populated by wild ungulates (*2*). Residents of these areas and forestry workers are at risk for Lyme disease (*3*). Heavily populated flat regions are not considered as risk areas. For example, *I. ricinus* ticks have never been reported in the flat areas of the Po River Valley in the Lombardy region, one of the most important industrial districts in Europe and an area of intensive agriculture and livestock breeding. Human population density is high; >6 million persons reside in Milano and surrounding counties. In areas of Italy to which *I. ricinus* ticks are known to be endemic, physicians have appropriate awareness of the risks from tick bite and Lyme disease; outside these areas, awareness probably is not adequate.

In late spring 2008, a forestry worker at a natural park west of Milano in the Po River Valley was treated for cutaneous mycosis on the basis of an erythematous rash on an arm. In August 2008, this patient described this skin alteration to one of us (C.B.). Subsequent clinical examination and serologic analyses led to diagnosis of Lyme disease. Because of this case, we investigated different areas of the park for ticks; collected ticks were screened by PCR for Lyme borreliae. We conducted a retrospective analysis of forestry workers in the area; 2 workers reporting the appearance of erythematous rash in the previous months underwent serologic analyses.

## The Study

During May–August 2009, ticks at different stages of development were collected by dragging. Ticks were collected in rural or suburban areas of the municipalities of Somma Lombardo, Lonate Pozzolo, Magenta, and Pavia (Figure). These sites are located along the Ticino River, which crosses the counties of Varese, Novara, Milano, and Pavia.

**Figure:**
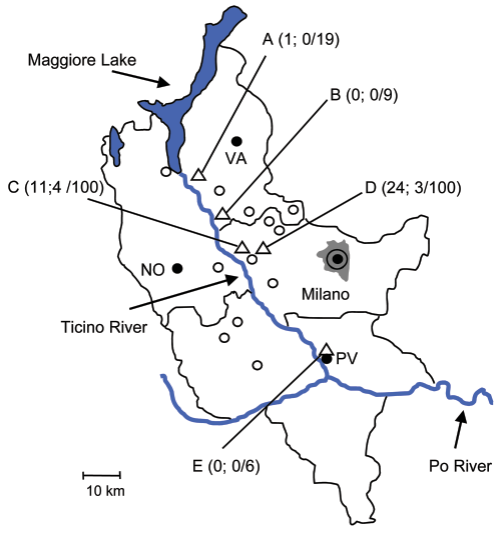
Collection sites (triangles A–E) of *Ixodes ricinus* ticks in the counties of Milano, Pavia, and Varese, Po River Valley, Italy, 2008. Ticks were collected in rural or suburban areas of the municipalities of Somma Lombardo (collection site A), Lonate Pozzolo (B), Magenta (C, D), and Pavia (E). The 3 numbers in parentheses for each collection site indicate number of tick nymphs positive for *Borrelia afzelii,* number of nymphs positive for *B. lusitaniae*, and number of nymphs examined by PCR. The adult specimen positive for *B. afzelii* was collected at site D. Empty circles indicate towns with 10,000–50,000 residents; black circles indicate towns with >50,000 residents. NO, Novara; PV, Pavia; VA, Varese. Milano residents = 4 million persons.

Of 1,094 ticks collected, 576 were larvae, 507 nymphs, and 11 adults (7 males). The samples were stored in 96% ethanol and later identified according to standard taxonomic keys (*4*). All ticks were *I. ricinus*. In a subsample of 240 nymphs of the 507 collected, each nymph was broken apart with a sterile needle and then subjected to DNA extraction by using the IllustraTissue & Cells Genomic Prep Mini Spin Kit (GE Healthcare, Little Chalfont, UK). The quality of the extracted DNA was checked by PCR for the *I. ricinus* tick mitochondrial 12S rRNA gene (*5*). Positive amplification was found for most (234/240) nymphs examined. A subset of the amplified 12S rRNA genes (20/234) were sequenced by using ABI technology (Applied Biosystems, Foster City, CA, USA), and the sequences obtained confirmed the specimens as *I. ricinus*. PCR screening for *B. burgdorferi* sensu lato was performed on DNA from the 234 nymphs by using primers BBLD5′ and BBLD3′ for 16S rRNA (*6*). Positive samples were examined by using a nested PCR protocol (*7*) for the 23S–5S rRNA spacer region of *B. burgdorferi* sensu lato. In addition, all 11 adults and pools of 10 larvae from Somma Lombardo, Lonate Pozzolo, Magenta (collection sites A, B, and C in Figure) were screened for *B. burgdorferi* sensu lato by using the same procedure.

*B. burgdorferi* sensu lato was detected in 42 (18%) of the 234 nymphs analyzed (Figure). One of the 7 adult males was positive; none of the 4 adult females and none of the pools of larvae were positive. The PCR products obtained from the 42 positive nymphs and from the adult male were sequenced by using ABI technology (Applied Biosystems), and the sequences were searched for homology using BLAST on the National Center for Biotechnology Information nonredundant database (www.ncbi.nlm.nih.gov/BLAST). 16S rRNA sequences confirmed identification as *B. burgdorferii* sensu lato, whereas rRNA spacer sequences showed the highest scores for *B. afzelii* (36/43) and *B. lusitaniae* (7/43) (Figure). Six rRNA spacer sequences representing the entire variability were deposited in the European Molecular Biology Laboratory database (FN658703–FN658708), then aligned with homologous sequences of *Borrelia* species by using MUSCLE (*8*). Neighbor-joining phylogenetic analysis, using SeaView 4.2 (*9*), confirmed placements of the obtained sequences into the clusters of *B. afzelii* and *B. lusitaniae* (data not shown). The relative prevalence of the 2 species of borreliae that we detected differs from those reported in other studies (*10*), probably because of environmental conditions, particularly the presence and relative abundance of the different reservoir hosts (*11*).

The 3 forestry workers who reported having had cutaneous erythema, 2 of whom noted a tick bite, underwent physical examination, then ELISA and Western blot analysis following the recommended stepwise protocol for the serodiagnosis of Lyme disease (*12*). Western blot, performed by using the recomBlot Borrelia IgG kit (Mikrogen Diagnostik, Neuried, Germany), according to the manufacturer’s instructions, confirmed the positive results of the ELISA. The 3 patients then received the recommended therapy (*13*).

## Conclusions

We found evidence of the Lyme disease vector *I. ricinus* ticks in the Po River Valley in the Lombardy region of Italy and of ticks from 3 locations that harbored Lyme disease borreliae. In addition, we detected evidence for *B. burgdorferi* sensu lato infection in 3 persons at risk for tick bite who work in the area. One location from which we collected *I. ricinus* ticks (location E) is in the suburban area of Pavia, a densely populated town. The risk of contracting Lyme disease in Italy is thus not limited to mountains and wild areas but extends to the plains, such as the Po River Valley, and possibly reaches suburban areas. The characteristics of the territory of the sampled area, although in heavily populated counties, are ecologically compatible with the presence of *I. ricinus* ticks because of the woods and bushes, corridors of vegetation connecting the plains and the river banks to mountain areas, and microrodents. In addition, the area along the Ticino River that includes collection locations C and D (where most tick specimens were sampled) is populated by roe deer (*Capreolus capreolus*), whose role as a major host for *I. ricinus* ticks is well known (*2*). These ungulates were introduced into this area 2 decades ago (*14*).

The Lyme disease case initially diagnosed as a mycosis and the 2 undiagnosed cases among forestry workers in the area west of Milano suggest that awareness of risks associated with tick bite probably is not adequate among physicians in the region. Moreover, before our investigation, visitors of the wild areas along the River Ticino were not adequately informed about the presence of ticks. Our report provides a basis for supplying proper information to health institutions and physicians in the area, as well for helping park administrators adopt proper precautions.
